# Editorial: Gut-lung axis: tuberculosis and drug resistance

**DOI:** 10.3389/fmicb.2024.1469562

**Published:** 2024-08-12

**Authors:** Samira Tarashi, Kianoosh Ferdosnejad, Abolfazl Fateh, Seyed Davar Siadat

**Affiliations:** ^1^Microbiology Research Center (MRC), Pasteur Institute of Iran, Tehran, Iran; ^2^Department of Mycobacteriology and Pulmonary Research, Pasteur Institute of Iran, Tehran, Iran; ^3^Department of Biology, Science and Research Branch, Islamic Azad University, Tehran, Iran

**Keywords:** *Mycobacterium tuberculosis*, microbiota, gut-lung axis, antibiotics, antimicrobial resistance

The gut microbiota, consisting of microorganisms in the digestive tract, plays a key role in maintaining overall health. Researchers have found connections between this ecosystem and different organs and systems, impacting immunity, metabolism, and functions. Among these connections, the gut-lung axis stands out as a complex area with potential to transform treatment of tuberculosis (TB) and drug resistance. Moreover, the gut microbiota affects the metabolism of TB drugs. *Mycobacterium tuberculosis* infections are a global health issue requiring effective control measures, with the study of human microbiota playing a crucial role (Wu et al., 2023). Understanding how the gut microbiota, immune system, and drug metabolism are interconnected can lead to targeted therapies addressing the root issues of TB infection and resistance.

Imbalances in microbiota composition are significant factors in *M. tuberculosis* pathogenesis and drug metabolism. However, few studies have explored how *M. tuberculosis* infection interacts with gut-lung axis microbiota composition. This Research Topic aimed to uncover this connection, emphasizing the importance of the gut-lung axis and drug resistance in TB management strategies. The focus is on *M. tuberculosis*, but research on other *Mycobacteriaceae* strains is also included. Within this Research Topic, five articles have been published, expanding our understanding of the role of the gut-lung axis in TB infection and drug resistance.

Enjeti et al. examined the connections between TB infection and the gut-lung axis, highlighting the crucial role of gut microbiota in influencing lung immunity with signaling molecules like short-chain fatty acids (SCFAs). This interaction modulates inflammatory responses in disease conditions. The link between gut microbiota and lung immunity is suggested to be significantly associated with TB pathophysiology. Reduced species richness and diversity of gut microbiota in TB patients compared to healthy individuals suggest an impact on *M. tuberculosis* infection, potentially leading to gut damage allowing microbial infiltration and immune response activation (Pant et al.). These immune cells and molecules can travel to other body sites through the bloodstream. In the lungs, bacterial components trigger IFNβ release and Tregs activation, while microbial products dampen immune reactions. Specific organisms and metabolites exhibit anti-TB properties; for example, indole propionic acid and *Helicobacter pylori*'s metabolites are thought to hinder *M. tuberculosis* survival by promoting pro-inflammatory responses (Enjeti et al.).

In addition to the pathogenic links in TB infection and the gut-lung axis, various factors influence this relationship. This link may be differently impacted based on individual susceptibility to active TB and latent TB infection (LTBI), reactivation from latency, clearance with or without drugs, and genetic diversity of *M. tuberculosis*. These differences may stem from the varying structural and functional makeup of gut microbiota and subsequent inter-individual variations in metabolizing nutrient components, affecting the effects of certain metabolites on health. Alvarado-Peña et al. studied the interaction between microbiota composition and the gut-lung axis in disease and treatment. Antibiotic use in TB treatment can disturb the bacterial balance in the body, especially in organs like the gut and lungs, influencing interactions among immune cells, bacteria, and signaling molecules in these regions. Alterations in microbiota patterns from antibiotic treatment may impact TB treatment outcomes, such as complete recovery, reinfection, and relapse. It is essential to investigate whether anti-TB treatment-induced microbiota dysbiosis could enhance susceptibility to TB reactivation post-cure and the emergence of drug resistance (Pant et al.). Atavliyeva et al. explored the significance of genetic diversity in *M. tuberculosis* strains, particularly lineage 2/Beijing family, as a significant public health concern. These strains exhibit high virulence and transmissibility (Ferdosnejad et al., 2024). Evidence suggests that different *M. tuberculosis* lineages have varied virulence factors and immunogenic properties that may affect their interaction with gut microbiota, immune system development, and response to *M. tuberculosis* infection. The association between different *M. tuberculosis* lineages and gut microbiota dysbiosis is a critical research area that could offer insights into TB pathogenesis and potentially novel treatment approaches. Enjeti et al. proposed that probiotic supplementation may alleviate issues in TB infection. Probiotics can modulate immune responses, reduce inflammation, and enhance antibiotic effectiveness in bacterial infection treatment. For instance, *Lacticaseibacillus rhamnosus*, obtained from the vaginal flora of healthy females, impeded the growth of *M. tuberculosis* in a laboratory setting and exhibited effectiveness in eradicating both drug susceptible and resistant strains of *M. tuberculosis* within murine macrophages, all without causing cytotoxicity (Rahim et al., 2022). Therefore, incorporating probiotics into TB treatment may enhance outcomes and reduce drug resistance risks. On the other hand, graphene quantum dots (GQDs) are nanomaterials with unique properties that show promise in biomedical applications, including antimicrobial therapy. Studies indicate GQDs' potential in inhibiting pathogenic bacteria growth, including *M. tuberculosis*. GQDs disrupt bacterial cell membranes, inhibit biofilm formation, and boost conventional antibiotics' antimicrobial activity. Exploring GQDs' use as a potential treatment for *M. tuberculosis* infection, alone or combined with antibiotics or probiotics, could offer innovative strategies for combating TB and addressing drug resistance issues (Santarelli et al.).

In conclusion, by investigating the complex interactions between the gut microbiota, immune response, and drug metabolism, new strategies can be developed to tackle tuberculosis and protect public health on a global scale. Targeted interventions such as probiotics, microbiota transplantation, and the use of various nanomaterials show potential in improving treatment outcomes and controlling the transmission of drug-resistant strains of *M. tuberculosis*. Looking toward the future, exploring the connection between the gut and lungs opens up exciting possibilities for further research and innovation in the fight against TB infection and drug resistance. By capitalizing on this dynamic relationship, it can be moved closer to the goal of eradicating *M. tuberculosis* infection as a significant global health threat.

## Author contributions

ST: Conceptualization, Validation, Writing – review & editing, Visualization, Writing – original draft. KF: Investigation, Writing – original draft. AF: Validation, Visualization, Writing – review & editing. SS: Supervision, Validation, Visualization, Writing – review & editing.
